# Transperineal Laser Ablation for Focal Therapy of Localized Prostate Cancer: 12-Month Follow-up Outcomes from a Single Prospective Cohort Study

**DOI:** 10.3390/cancers16152620

**Published:** 2024-07-23

**Authors:** Valerio Iacovelli, Marco Carilli, Riccardo Bertolo, Valerio Forte, Matteo Vittori, Beatrice Filippi, Giulia Di Giovanni, Chiara Cipriani, Filomena Petta, Francesco Maiorino, Marta Signoretti, Michele Antonucci, Alessio Guidotti, Stefano Travaglia, Francesco Caputo, Guglielmo Manenti, Pierluigi Bove

**Affiliations:** 1 Urology Unit, San Carlo di Nancy Hospital, GVM Care and Research, 00165 Rome, Italy; 2 Department of Urology, AOUI Verona, University of Verona, 37129 Verona, Italy; 3 Radiology Unit, San Carlo di Nancy Hospita, GVM Care and Research, 00165 Rome, Italy; 4 Department of Biomedicine and Prevention, Radiology Unit, Tor Vergata University of Rome, 00133 Rome, Italy

**Keywords:** prostate cancer, focal therapy, TPLA

## Abstract

**Simple Summary:**

Prostate cancer is the second most diagnosed cancer in men. Focal laser ablation has been proposed as an alternative to radical treatments in carefully selected patients in order to achieve long-term cancer control and reduce the morbidity associated with surgery and radiation therapy. This study aimed to evaluate the oncological and functional outcomes of transperineal laser ablation (TPLA) as the focal therapy for localized prostate cancer. We assessed that TPLA is a safe, painless, and effective technique with good oncological results and the preservation of continence and sexual outcomes.

**Abstract:**

Introduction and objectives: To evaluate the oncological and functional outcomes of transperineal laser ablation (TPLA) as the focal therapy for localized prostate cancer (PCa) after a 12-month follow-up. Materials and methods: Patients with low- and intermediate-risk localized PCa were prospectively treated with focal TPLA between July 2021 and December 2022. The inclusion criteria were the following: clinical stage < T2b; PSA < 20 ng/mL; International Society of Urological Pathology (ISUP) grade ≤ 2; MRI-fusion biopsy-confirmed lesion classified as PI-RADS v2.1 ≥ 3. Intra-, peri-, and post-operative data were collected. Variables including age, PSA, prostate volume (PVol), Charlson’s Comorbidity Index (CCI), International Prostate Symptom Score (IPSS) with QoL score, International Index of Erectile Function (IIEF-5), International Consultation on Incontinence Questionnaire—Short Form (ICIQ-SF), and Male Sexual Health Questionnaire—Ejaculatory Dysfunction Short Form (MSHQ-EjD) were collected at baseline and at 3, 6 and 12 months after TPLA. Post-operative mpMRI was performed at 3 and 12 months. Finally, all patients underwent prostatic re-biopsy under fusion guidance at 12 months. The success of this technique was defined as no recurrence in the target treated lesion at the 12-month follow up. Results: Twenty-four patients underwent focal TPLA. Baseline features were age [median 67 years (IQR 12)], PSA [5.7 ng/mL (3.9)], PVol [49 mL (27)], CCI [0 (0)], IPSS [11 (9)], IPSS-QoL [2 (2)], IIEF-5 [21 (6)], ICIQ-SF [0 (7)], MSHQ-EjD ejaculation domain [14 (4)] and bother score [0 (2)]. Median operative time was 34 min (IQR 12). Median visual analogue scale (VAS) 6 h after TPLA was 0 (IQR 1). The post-operative course was regular for all patients, who were discharged on the second post-operative day and underwent catheter removal on the seventh post-operative day. No patient had incontinence at catheter removal. A significant reduction in PSA (*p* = 0.01) and an improvement in IPSS (*p* = 0.009), IPSS-QoL (*p* = 0.02) and ICIQ-SF scores (*p* = 0.04) compared to baseline were observed at the 3-month follow-up. Erectile and ejaculatory functions did not show any significant variation during the follow-up. No intra- and peri-operative complications were recorded. Three Clavien–Dindo post-operative complications were recorded (12%): grade 1 (two cases of urinary retention) and grade 2 (one case of urinary tract infection). At the 12-month follow-up, eight patients showed mpMRI images referable to suspicious recurrent disease (PIRADS v2.1 ≥ 3). After re-biopsy, 7/24 patients’ (29%) results were histologically confirmed as PCa, 3 of which were recurrences in the treated lesion (12.5%). The success rate was 87.5%. Conclusions: The focal TPLA oncological and functional results seemed to be encouraging. TPLA is a safe, painless, and effective technique with a good preservation of continence and sexual outcomes. Recurrence rate at 12 months was about 12.5%.

## 1. Introduction

Prostate cancer (PCa) is the second most diagnosed cancer in men, with an estimated 1.4 million diagnoses worldwide in 2020 [1]. According to the latest European Association of Urology (EAU) guidelines on PCa, patients with localized disease classified as low- or intermediate risk of biochemical recurrence (BCR) can be managed either with active surveillance (AS) or treatment with curative intent, such as radical prostatectomy (RP) or external beam radiotherapy (EBRT) [2]. As shown by the ProtecT trial, active monitoring in clinically localized PCa provides a 10-year cancer specific survival of 99% [3]. Interestingly, a 5% rate of AS discontinuation was described due to anxiety and distress for untreated PCa [4].

In a recent Delphi consensus panel, focal laser ablation was proposed as an alternative to radical treatment in carefully selected patients, with a Gleason score (GS) ≤ 4 + 3 and magnetic resonance imaging (MRI)-visible, biopsy-proven PCa [5]. As previously described for benign prostatic hyperplasia (BPH) treatment, SoracteLite^TM^ transperineal laser ablation (TPLA) is a minimally invasive technique which aims to achieve the ablation of prostatic tissue through thermo-coagulation [6,7,8]. To date, focal TPLA has been evaluated only in small case series, appearing to be safe and feasible [9,10]. The aim of this study was to evaluate the short-term oncological and functional outcomes of focal TPLA.

## 2. Patients and Methods

### 2.1. Study Design

This study is a prospective, single center, interventional pilot study. The study was conducted in accordance with the Declaration of Helsinki and its later amendments. The study was approved by the local institutional ethics committee (n. approval STS CE Lazio 1/N-822) and has been registered on ClinicalTrials.gov as (NCT05584787).

### 2.2. Patients

From July 2021 to December 2022, patients with low- and intermediate-risk localized PCa who expressed an interest in focal therapy were enrolled in this study and written informed consent was obtained.

All patients received combined systematic (10–14 cores) and target (3–4 cores) transperineal prostate biopsy under MRI-US fusion guidance (BK Medical S.r.l., Melegnano, Italy). Inclusion criteria were the following: clinical stage < T2; PSA < 20 ng/mL; International Society of Urological Pathology (ISUP) grade ≤ 2; MRI-fusion biopsy-confirmed lesion classified as Prostate Imaging—Reporting and Data System (PI-RADS) v2.1 ≥ 3. Patients who had undergone previous pelvic radiotherapy and/or hormonal therapy, had a history of other genitourinary malignancies, multifocal disease or a tumor diameter ≥ 2 cm on multiparametric MRI (mpMRI), or had no MRI data available were excluded. Criteria are summarized in Table 1.

### 2.3. Outcome Measurements

Preoperative variables, including age, comorbidities assessed by Charlson’s Comorbidity Index (CCI), serum prostate specific antigen (PSA), prostate volume, International Prostate Symptom Score (IPSS) with Quality of Life (IPSS-QoL) score [11], International Index of Erectile Function (IIEF-5) [12], International Consultation on Incontinence Questionnaire—Short Form (ICIQ-SF) [13], and Male Sexual Health Questionnaire—Ejaculatory Dysfunction Short Form (MSHQ-EjD) [14] were collected at baseline.

Intra- and perioperative data were collected, including operative time and visual analogue scale (VAS) assessed 6 h after TPLA. Eventual post-operative complications were recorded and classified according to the Clavien–Dindo system [15]. Post-operative urinary incontinence was defined as patient requiring ≥1 pad after TPLA. Readmissions were recorded as well.

Follow-up visits were scheduled at 3, 6 and 12 months, including serum PSA and re-administration of standardized questionnaires.

Post-operative mpMRI was performed at 3 and at 12 months. Finally, all patients underwent prostatic re-biopsy under fusion guidance at 12 months, with at least 3 cores on the treated area. Success of this technique was defined as no recurrence in the target treated lesion at 12 months. Post-operative mpMRI were re-evaluated using the Transatlantic Recommendations for Prostate Gland Evaluation with Magnetic Resonance Imaging After Focal Therapy (TARGET) score [16].

### 2.4. Focal TPLA Procedure

TPLA was performed in the lithotomy position. All procedures were performed under conscious sedation combined with local anesthesia of perineal skin and peri-prostatic nerve-block with Lidocaine 2% (20 mL). Antibiotic prophylaxis consisted of a single IV dose of Cefazoline 2 g.

**Figure 1:**
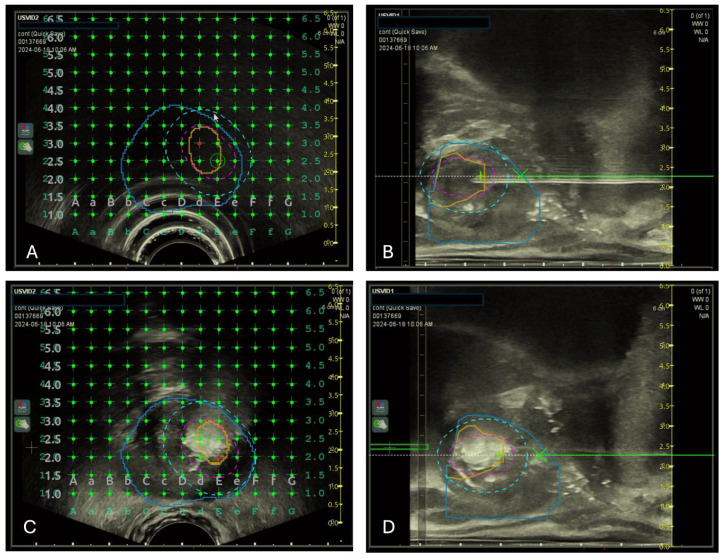
EchoLaser Smart Interface (ESI). Top row: (**A**) axial treatment planning. Prostate in blue with a target mpMRI identified lesion inside (orange). Pink dashed line identifies the area of treatment. Light blue dashed line identifies the safety margins. (**B**) longitudinal treatment planning. The big X indicates the tip of the needle; the + indicates the tip of the fiber. This planning allows the position of the fibers to best fit the volume and the shape of tumor lesion. Bottom row: (**C**,**D**) axial and longitudinal plans of ESI showing the limits of the treatment area (hyperechoic “bubbling” area) and the safety margins.

Focal TPLA was performed using the SoracteLite^TM^ EchoLaser EVO system (Elesta S.p.A., Calenzano, Italy). Similar to TPLA for BPH ablation [17], focal TPLA for PCa requires placement of optic fibers (Fiber Optic for PLA, Elesta S.p.A., Calenzano, Italy) through dedicated 21-gauge insertion needles (Introducer, Elesta S.p.A., Calenzano, Italy) inside the prostatic tissue. MRI-US fusion guidance (BK Medical S.r.l., Melegnano, Italy) was used to identify the focal lesion to treat. For the study, fiber placement was conducted under MRI-US fusion guidance. In addition, the device EchoLaser Smart Interface (ESI) allowed the user to pre-visualize and verify the positioning of the fibers, in order to best fit the volume and the shape of the tumor lesion. Through a system of concentric circles, ESI shows the borders of the treatment area, the extra safety margin to treat in order to minimize the risk that cancerous tissue might remain untreated, finally the safety area indicating critical anatomical sites to preserve from laser energy (Figure 1).

Depending on the target lesion volume, 1 or 2 fibers were placed transperineally and connected to a multisource laser system operating at 1.064 nm. Following introduction of the optical fiber through the needle, the needle was retracted for 10 mm, allowing exposure of the fiber tip and the first 10 mm of the fiber to the tissue. During a treatment session, 1800 J were delivered per fiber at 5 W. The ellipsoid area of coagulative necrosis for each fiber extends to 16–18 mm longitudinally (of which two-thirds is localized beyond the tip of the fiber and one-third behind the tip) and 10–12 mm transversely [18]. When 2 fibers were needed, they were activated simultaneously. If necessary, a “pullback” technique (which consists of retracting the fiber 1 cm along its trajectory to deliver more energy [1000–1800 J]) was performed to ensure complete cancer ablation. The laser therapy was performed entirely under the US guidance for the real-time monitoring of the correct positioning of applicators and the extension of the treated area.

All patients were discharged on 2nd post-operative day and underwent catheter removal on 7th post-operative day.

### 2.5. Statistical Analysis

Continuous variables were summarized using medians and interquartile ranges (IQR); frequencies and proportions were used to report categorical variables. Wilcoxon non-parametric test was performed to compare repeated measures over time. Chi-square test was used for categorical variables. Data analysis was conducted using SPSS 21.0 software (IBM, Armonk, NY, USA). Statistical significance was defined as *p*-value < 0.05.

## 3. Results

Twenty-four patients underwent focal TPLA. A total of 10 patients were excluded (2 who had ISUP grade ≥ 3, 6 who had multifocal disease on systematic prostate biopsy, 1 who had undergone previous pelvic radiotherapy, 1 who had a history of bladder cancer).

The clinical and demographic baseline characteristics of the study population are summarized in Table 2. Four patients (16.7%) personally chose to shift from AS to TPLA during follow-up; the remaining study population received TPLA as first choice active treatment instead of robot-assisted radical prostatectomy (RARP) or EBRT.

### 3.1. Peri-Operative, Post-Operative Outcomes and Complications

Peri-operative outcomes are reported in Table 3. Pullback was necessary in four cases (16.7%) to ensure complete cancer ablation. The post-operative course was regular for all patients. The procedure was almost painless, as suggested by VAS score. No patient developed post-operative complications or adverse events, and no readmission was required. At catheter removal, seven patients (29.2%) reported transient urgency (<72 h). No patient had incontinence at catheter removal.

### 3.2. Functional Outcomes

**Figure 2:**
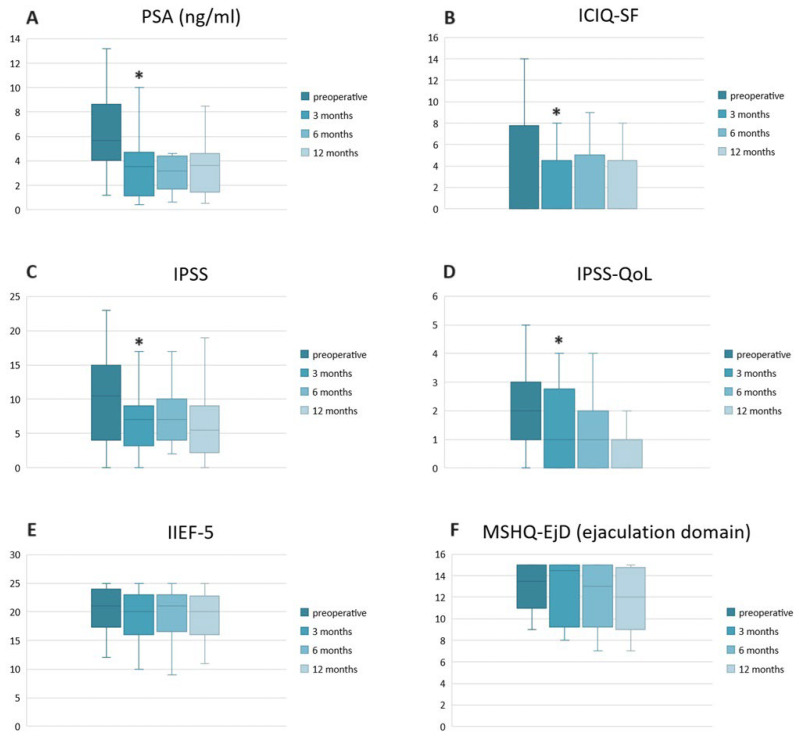
Graphic changes in serum PSA and questionnaire scores during follow-up. (**A**) Prostate specific antigen (PSA); (**B**) International Consultation on Incontinence Questionnaire—Short Form (ICIQ-SF); (**C**) International Prostate Symptom Score (IPSS); (**D**) IPSS-Quality of Life score (IPSS-QoL); (**E**) International Index of Erectile Function (IIEF-5); (**F**) Male Sexual Health Questionnaire—Ejaculatory Dysfunction Short Form (MSQH-EjD). The asterisk (*) indicates a statistically significant difference (*p* < 0.05).

An improvement in IPSS (*p* = 0.009), IPSS-QoL (*p* = 0.02) and ICIQ-SF scores (*p* = 0.04) compared to baseline was observed at the 3-month follow-up. Erectile and ejaculatory functions as assessed by IIEF-5 and MSHQ-EjD did not show any significant variation during the follow-up (*p* > 0.05) (Table 3, Figure 2).

### 3.3. Oncological Outcomes

At 3 months, the mpMRI showed complete ablation of the index lesion in all patients, an absence of images referable to residual disease in the treated area and/or newly diagnosed lesions. A significant reduction in PSA values (*p* = 0.01) was detected at 3 months (Table 3, Figure 2).

At 12 months, eight patients showed images referable to recurrent disease (three infield vs. five outfield of the previously treated lesion). When considering the TARGET score, the three infield images referable to recurrent disease of a previously treated lesion were classifiable as TARGET 5 (two cases) and TARGET 4 in one case. For the other patients, a complete response to TPLA was classified as TARGET 1 (18 cases) and 2 (3 cases).

After re-biopsy, seven patients (29.2%) were histologically confirmed as PCa recurrence (six patients ISUP grade group 1, and one patient ISUP grade group 2). Finally, three/seven cases were recurrences in the treated lesion (12.5%). Thus, the success rate at 12 months was 87.5%.

Patients diagnosed with PCa recurrence were finally scheduled for RARP (four patients), EBRT (two patients) or focal TPLA of new target lesion (one patient). None of these procedures showed any deviation from the standard post-operative course, and no post-procedural complication linked to previous focal TPLA was recorded.

## 4. Discussion

In recent decades, screening strategies have led to a rise in the detection of earlier stage PCa; thus, whole gland therapies may expose patients to overtreatment [2].

Historically, treatments involving the entire prostate gland were considered mandatory, given the multifocality of PCa (about 80% of cases). Nevertheless, evidence suggests that extraprostatic extension and BCR are determined by the so-called “index lesion” (i.e., the largest and least-differentiated tumor lesion) [19,20].

Focal therapy aims to ablate the index lesion alone through different energy sources, to achieve oncological outcomes like whole-gland definitive treatments while avoiding treatment-related toxicity and complications [21,22]. To date, a few ablative therapies have reported results on mainly low-risk disease [23]. Given the lack of data on long-term oncological outcomes, the available evidence indicates that focal therapy should not be performed outside a clinical trial setting or well-designed prospective cohort study [2].

This prospective trial was conceived to offer a “micro-invasive” treatment of PCa. Some advantages of TPLA over other ablative options are that tissue ablation is monitored in real time via MRI-US fusion guidance, the procedure is fast, there is the opportunity for re-treatment, and very few treatment-related complications are reported [5,6,7,8,9,10].

We did not report any adverse event related to TPLA on erection, ejaculation, and continence. Overall success rate, defined as no recurrence in the target, treated lesion at the 12-month re-biopsy, was 87.5%.

Our results seemed aligned with the available literature. In 2022, Van Riel et al. reported their first experience in 12 patients with localized PCa who were scheduled for RARP (“ablate and resect design”). The authors did not report any device-related adverse events, which were mostly related to lower urinary tract symptoms and were mild. Erectile function showed a mild impact and returned to baseline after 4 weeks, and TPLA treatment did not compromise RARP [9]. In 2023, the same group reported their 4-week post-TPLA follow-up experience with contrast-enhanced ultrasound (CEUS) and mpMRI that could reliably visualize TPLA ablative effects and did not show any cancer remnant [24].

In 2022, Meneghetti et al. reported their preliminary experience with focal TPLA in 10 patients. After 6 months, persistent disease was detected in three patients (30%) who underwent a second ablation that did not harbor residual disease. The authors did not report any incontinence cases, worsening in sexual potency nor prostatic symptoms measured at IPSS at the 12-month follow-up. PSA was significantly reduced at 12 months vs. baseline [10].

Very few studies report oncological outcomes following focal laser ablation (FLA) beyond 1 year [25]. Interestingly, Chao et al. reported data on QoL and in-field recurrence following FLA on 32 patients. The authors reported that FLA did not impact urinary or sexual function and that MRI is a proper tool able to identify in-field recurrence in intermediate- and low-risk PCa at 2 years after FLA without a significant changes in PSA from baseline [26].

Offering a focal TPLA for PCa imposed a strict follow-up protocol, including digital rectal examination and PSA (at 3, 6 and 12 months), mpMRI (at 3 and 12 months) and repeated biopsy at 12 months. Proper follow-up schedules are mandatory for this new technique. Recently, Cocci et al. evaluated TPLA with EchoLaser using the Delphi consensus method. The Delphi panelists recommended TPLA for unilateral and small low- and intermediate-risk PCa in any localization of the gland. Regarding the outcomes, mpMRI at 3 months has been considered the standard restaging technique after TPLA. The Delphi defined a treatment success as both negative mpMRI and random biopsy of the treatment area [27].

In the light of these preliminary and promising results, even the functional outcomes should be considered. In all the papers reported, both ejaculatory function and external urethral sphincter function were preserved [28]. Regarding continence, TPLA seems to offer advantages over RARP. In fact, to highlight the impact of radical surgery on continence recovery, several technical refinements (e.g., bladder-neck sparing, Rocco stitch, etc.) and preoperative anatomical features (e.g., prostatic apical shape, membranous urethral length, etc.) [29] have been described. In contrast, TPLA’s impact on continence seems not to be related to any specific anatomical feature;: in fact, the treated lesion’s site does not affect functional outcomes.

**Figure 3:**
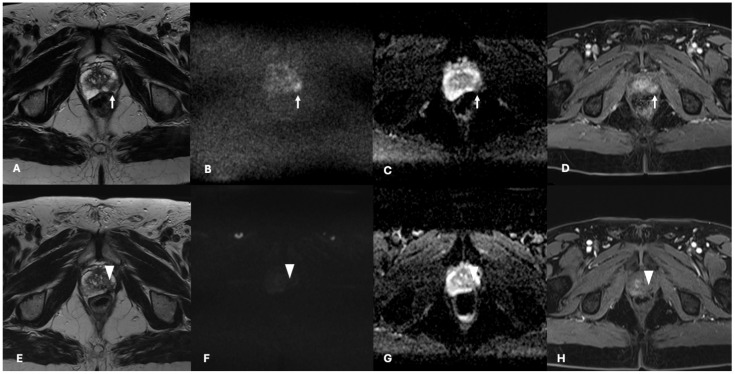
mpMRI assessment of lesions within the ablation zone after TPLA: a case of complete response. Top row ((**A**) T2 axial imaging, (**B**) DWI b = 1400 s/mm^2^, (**C**) ADC map, (**D**) dynamic contrast imaging) shows a PI-RADS 4 lesion (11 × 4 mm—white arrow) in the lateral aspect of the left peripheral zone of the prostate. Biopsy confirmed to be a Gleason Grade 7 (3 + 4), ISUP 2, treated with TPLA focal therapy. Bottom row ((**E**) T2 axial imaging, (**F**) DWI b = 1400 s/mm^2^, (**G**) ADC map, (**H**) dynamic contrast imaging) at 12-month follow-up; no residual cancer (white arrowhead) was detected, thus the score for this area is TARGET 1.

**Figure 4:**
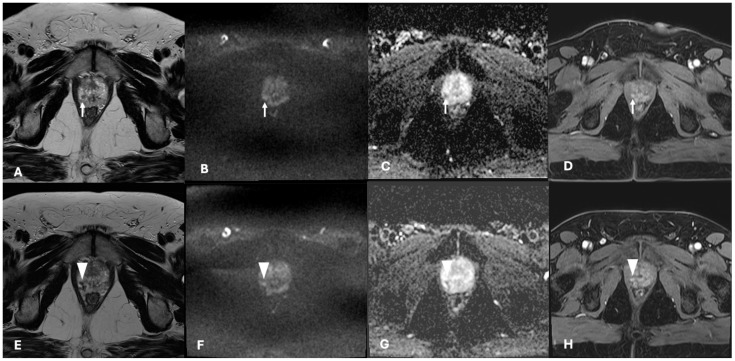
mpMRI assessment of lesions within the ablation zone after TPLA: a case of residual tumor. Top row ((**A**) T2 axial imaging, (**B**) DWI b = 1400 s/mm^2^, (**C**) ADC map, (**D**) dynamic contrast imaging) highlights a PI-RADS 4 lesion (5 mm—white arrow) located in the antero-lateral aspect of the right peripheral zone, histologically proven to be a Gleason 6 (3 + 3), ISUP 1. Patient underwent focal therapy; however, after 12 months, MRI shows a small focus (white arrowhead) proximal to the treated area ((**E**) T2 axial imaging, (**F**) DWI b = 1400 s/mm^2^, (**G**) ADC map, (**H**) dynamic contrast imaging), in keeping with residual cancer, classified as TARGET 5.

Recently, Light et al. presented a consensus on the Transatlantic Recommendations for Prostate Gland Evaluation with MRI after Focal Therapy (TARGET) to provide minimum standards for study reporting [16]. The TARGET score is a two-tier algorithm which incorporates a major dynamic contrast enhancement (DCE) sequence and joint minor (diffusion-weighted imaging) DWI and T2W sequences. After assessing the sequences, an overall score out of 5 was calculated as TARGET 1 = very low suspicion; 2 = low suspicion; 3 = equivocal; 4 = high suspicion; and 5 = very high suspicion [16]. A retrospective analysis of our cases showed that the three infield images referable to recurrent disease of previously treated lesion were classifiable as TARGET 5 (two cases) and TARGET 4 in one case. For the other patients, a complete response to TPLA was classified as TARGET 1 (18 cases) and 2 (3 cases). The mpMRI assessment of focal treatment results following the TARGET system is reported in Figure 3 and Figure 4.

Nowadays, there is lack of well-powered comparison studies between TPLA and other focal techniques.

Pilot studies reported results in low-risk PCa using the Indigo Optima FLA system using one or two laser sources, operating at 830 nm wavelength under general anesthesia or the Visualase system, which operates at 980 nm wavelength which only allows for single fiber treatment, and requires fiber replacement to create a larger ablation zone and subsequently longer treatment duration [30,31]. van Riel et al. reviewed these FLA systems that showed safety, feasibility, and stable short-term functional outcomes [9]. The authors suggested a comparison with Soractelite™ TPLA treatment using the Echolaser^®^ device which seemed to show some potential advantages: 1. US/MRI fusion guidance allows a real, minimally invasive treatment performable even under local anesthesia, in an outpatient setting, without an anesthesiologist and with potentially cost-effective results; 2. Echolaser^®^ works with four independently adjustable continuous wave laser diodes that may improve the shaping and the volume-dependent treatment of the ablation zone. Moreover, Echolaser^®^ operates at a 1064 nm wavelength, which has increased penetration depth, in comparison with the lasers operating at 830 or 980 nm [9].

Furthermore, when considering transrectal ablation, such as that described by Walser et al. [32], all FLA procedures were carried out in the Siemens 3T Skyra MRI unit with longer median procedure time [median procedure time was 122 min (60–250 min) vs. ours of 34 min (IQR 12)]. A single fiber was used and repositioned multiple times. Finally, following the European Association of Urology on prostatic biopsy [2], the transperineal approach should be preferred. Thus, as Patelli et al. reported, TPLA performed under US guidance has the comparative advantages of shorter total procedural times, lower risk of infection (no potential for prostatic inoculation by rectal bacteria), and no need for costly access to an MR imaging scanner. In contrast, one possible advantage of the MR approach is the real-time MR thermometry that allows thermal monitoring of the ablation zone [33].

We acknowledge the limitations of the study. First, the small sample size and short follow-up. Second, TPLA requires a surgeon skilled in the transperineal approach, which is still uncommon. Given our consistent experience in the transperineal approach, these results could not be fully reproducible. Last, there is still a lack of proper radiological methods to describe the effects of TPLA on prostatic tissue and to detect eventual PCa relapses even though the role of mpMRI is relevant [34]. The TARGET score is still underused.

Long-term follow up data is missing along with its standardized protocol. After the first 12 months, we follow our patients through the PRIAS schedule on active surveillance (Prostate cancer Research International: Active Surveillance, [35] and its updates on https://prias-project.org/modules/articles/article.php?id=3, accessed on 18 July 2024). which consists of PSA testing every three months in the first two years and every six months thereafter. At 6, 12, 18, and 24 months, and annually thereafter, evaluation visits will be scheduled with a physical examination and a digital rectal examination. Additionally, after 1, 4, 7, and 10 years, and every five years thereafter, a repeat biopsy is scheduled, possibly combined with an MRI and targeted biopsies [35].

Notwithstanding those limitations, TPLA is a safe and effective procedure. In selected patients, it could represent a “more” active surveillance, with the possibility to “precisely” treat the tumor, avoiding complications.

## 5. Conclusions

TPLA is a safe micro-invasive treatment for low- and intermediate-risk PCa that could offer good oncological and functional results. Higher-powered and better designed studies may investigate the relative role of this technique in comparison with active surveillance or radical treatments.

## Figures and Tables

**Table 1:** Clinical and demographic baseline patients characteristics.

	N = 24
Age (yr), median (IQR)	67 (12)
CCI, median (IQR)	0 (0)
Previous prostate surgery, n (%)	
No	20 (83.3)
TURP	3 (12.5)
HoLEP	1 (4.2)
Previous active surveillance, n (%)	
No	20 (83.3)
Yes	4 (16.7)
Preoperative PSA (ng/mL), median (IQR)	5.7 (3.9)
Prostate volume (mL), median (IQR)	49 (27)
PI-RADS, n (%)	
3	4 (16.7)
4	17 (70.8)
5	3 (12.5)
Site of PI-RADS lesion, n (%)	
Posterior zone	9 (37.5)
Anterior zone/transitional zone	6 (25.0)
Apical zone	9 (37.5)
Size of PI-RADS lesion (mm), median (IQR)	12 (5)
ISUP grade group, n (%)	
1	14 (58.3)
2	10 (41.7)

IQR interquartile range, CCI Charlson’s Comorbidity Index, TURP transurethral resection of prostate, HoLEP holmium laser enucleation of prostate, PSA prostate specific antigen, PI-RADS Prostate Imaging—Reporting and Data System, ISUP International Society of Urological Pathology.

**Table 2:** Intra- and peri-operative outcomes.

	N = 24
Operative time (min), median (IQR)	34 (12)
Laser ablation time (min), median (IQR)	12 (2)
N° of needles, n (%)	
One needle	18 (75.0)
Two needles	6 (25.0)
Pullback, n (%)	4 (16.7)
6 h post-operative VAS, median (IQR)	0 (1)
Post-operative complications, n (%)	0 (0)
Hospital readmissions, n (%)	0 (0)

IQR interquartile range, VAS visual analogue scale.

**Table 3:** Results of serum PSA and questionnaire scores during follow-up.

N = 24	Baseline	3 Months	*p*-Value ^a^	6 Months	*p*-Value ^b^	12 Months	*p*-Value ^c^
PSA (ng/mL), median (IQR)	5.7 (3.9)	3.6 (3.1)	0.01	3.2 (2.2)	0.5	3.7 (2.7)	0.9
IPSS, median (IQR)	11 (9)	7 (5)	0.009	7 (6)	0.7	6 (6)	0.3
IPSS-QoL, median (IQR)	2 (2)	1 (2)	0.02	1 (2)	0.8	1 (1)	0.1
IIEF-5, median (IQR)	21 (6)	20 (7)	0.2	21 (6)	0.5	20 (6)	0.8
ICIQ-SF, median (IQR)	0 (7)	0 (4)	0.04	0 (3)	0.8	0 (4)	0.9
MSHQ-EjD (ejaculation domain), median (IQR)	14 (4)	15 (5)	0.4	13 (5)	0.9	12 (5)	0.5
MSHQ-EjD (bother item), median (IQR)	0 (2)	0 (2)	0.5	1 (2)	0.9	0 (0)	0.07

^a^ *p*-value between 3-month follow-up and baseline. ^b^ *p*-value between 6-month and 3-month follow-up. ^c^ *p*-value between 12-month and 6-month follow-up. IQR interquartile range, PSA prostate specific antigen, IPSS International Prostate Symptom Score, IPSS-QoL IPSS-Quality of Life score, IIEF-5 International Index of Erectile Function, ICIQ-SF International Consultation on Incontinence Questionnaire—Short Form, MSHQ-EjD Male Sexual Health Questionnaire—Ejaculatory Dysfunction Short Form.

## Data Availability

The raw data supporting the conclusions of this article will be made available by the authors upon request.
